# Automated Mesiodens Classification System Using Deep Learning on Panoramic Radiographs of Children

**DOI:** 10.3390/diagnostics11081477

**Published:** 2021-08-15

**Authors:** Younghyun Ahn, Jae Joon Hwang, Yun-Hoa Jung, Taesung Jeong, Jonghyun Shin

**Affiliations:** 1 Department of Pediatric Dentistry, School of Dentistry, Pusan National University, Yangsan 50612, Korea; ahn7750@pusan.ac.kr (Y.A.); tsjeong@pusan.ac.kr (T.J.); 2 Department of Oral and Maxillofacial Radiology, School of Dentistry, Pusan National University, Yangsan 50612, Korea; softdent@pusan.ac.kr (J.J.H.); yhjung@pusan.ac.kr (Y.-H.J.); 3 Dental and Life Science Institute & Dental Research Institute, School of Dentistry, Pusan National University, Yangsan 50612, Korea

**Keywords:** mesiodens, artificial intelligence, deep learning, convolutional neural networks

## Abstract

In this study, we aimed to develop and evaluate the performance of deep-learning models that automatically classify mesiodens in primary or mixed dentition panoramic radiographs. Panoramic radiographs of 550 patients with mesiodens and 550 patients without mesiodens were used. Primary or mixed dentition patients were included. SqueezeNet, ResNet-18, ResNet-101, and Inception-ResNet-V2 were each used to create deep-learning models. The accuracy, precision, recall, and F1 score of ResNet-101 and Inception-ResNet-V2 were higher than 90%. SqueezeNet exhibited relatively inferior results. In addition, we attempted to visualize the models using a class activation map. In images with mesiodens, the deep-learning models focused on the actual locations of the mesiodens in many cases. Deep-learning technologies may help clinicians with insufficient clinical experience in more accurate and faster diagnosis.

## 1. Introduction

Supernumerary teeth are defined as the teeth that exceed the normal number of teeth. The cause of excessive number of teeth has not been clearly identified, and both genetic and environmental factors are presumed to be involved. Supernumerary teeth are most common in the midline of maxilla and are called mesiodens [1]. The prevalence of mesiodens has been reported to be 0.1–7.0% [2].

Mesiodens can cause various complications, such as dentigerous cysts, resorption of the roots of adjacent permanent teeth, eruption disorders of the maxillary incisors, diastema, and crowding. If inverted mesiodens are not detected early and left neglected, these may move towards the nasal cavity, increasing the difficulty in performing the surgical operation [3]. Therefore, to prevent such complications, it is imperative to detect the mesiodens in advance, and to extract them at an appropriate time.

If cone-beam computed tomography (CBCT) is used for the diagnosis of mesiodens, the precise shape and comprehensive location information of the impacted mesiodens can be easily identified in three dimensions [4]. However, children are more sensitive to radiation than adults are, and when radiographs of children are taken under the same conditions as those for adults, the risk of exposure is high [5]. For this reason, CBCT cannot be performed on a regular basis. It is necessary to evaluate the justification of exposure of radiation on children before using CBCT [6]. However, panoramic radiographs provide considerable diagnostic information within the jaw with a single radiograph and are often used in dentistry because they offer the advantage of relatively low radiation exposures, compared to those with CBCT [7]. Even in children, panoramic radiographs are often taken for evaluating the stage of tooth development and dental caries. However, the panoramic radiographs of patients with mixed dentition are complicated owing to the mixture of primary and successive permanent teeth; thus, there are several factors that dentists must carefully consider. Additionally, owing to the difference in size of the maxillary arch between children and adults, the image of the upper anterior region of a child is not accurately included in the focal trough [4]. Owing to these difficulties, dentists unfamiliar with mixed dentition panoramic radiographs may miss important diagnostic information [8].

Currently, the deep convolutional neural network (DCNN) technology is being actively applied in the field of dental imaging [9]. Artificial intelligence has been reported to excellently judge various diseases in the oral and maxillofacial areas [10,11,12,13]. With the introduction of class activation map technology, the black-box effect of deep-learning models has been overcome, and judgments can be visualized [14]. However, only a few studies have used artificial intelligence for diagnosing mesiodens, and each study on patients with mixed dentition has a limitation: only a single deep-learning model has been used [15,16]. To the best of our knowledge, there has not been any study that has visualized the decision of the deep-learning model using the class activation map to locate the mesiodens.

Therefore, this study aimed to use several deep-learning networks to create models that automatically detect mesiodens in panoramic radiographs of children with mixed dentition, evaluate the performance of each model, and visualize the model using the class activation map.

## 2. Materials and Methods

### 2.1. Ethics Statement

This study was conducted with the approval of the Institutional Review Board (IRB) of Pusan National University Dental Hospital (IRB No.: PNUDH-2020-006).

### 2.2. Subjects

This study was conducted on panoramic and CBCT radiographs of Pusan National University Dental Hospital patients from January 2013 to January 2020. Patients who were diagnosed with one or more mesiodens via CBCT were selected as the experimental group. As controls, dental age-matched patients without mesiodens were selected from the same database (Table 1). CBCT (Pax-Zenith3D; Vatech Co., Ltd., Hwaseong, Korea) was performed with the following scanning parameters: 105 kVp, 4 mA, 24 s, voxel size of 0.2 mm, and field of view of 20 × 19 cm. All patients included in this study were in Hellman’s dental developmental stages IIA, IIC, and IIIA [17]. Panoramic radiographs, which were difficult to interpret owing to severe distortions of images, and patients with developmental tooth disorders or orthodontic treatment were excluded. Panoramic radiographs of 550 patients in the experimental group and 550 patients in the control group were used in the study.

### 2.3. Methods

#### 2.3.1. Data Preprocessing

All panoramic radiographs were taken using a Proline XC machine (Planmeca Co., Helsinki, Finland) and downloaded as JPEG files (2943 × 1435 pixels).

**Figure 1:**
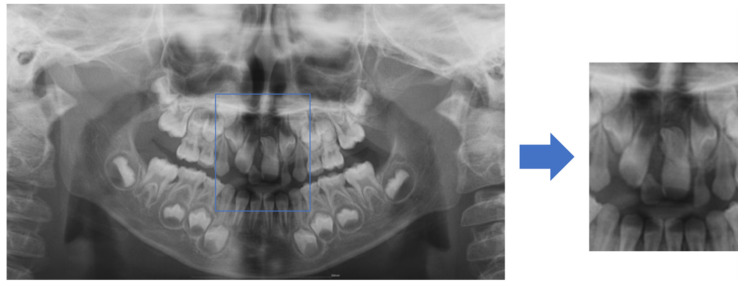
Region of interest (ROI). Images were cropped, as shown in blue box, based on the distal and uppermost points of both permanent canine tooth germs and mandibular anterior alveolar bone level.

To increase the mesiodens detection accuracy of the deep-learning models, the anterior region was set as the region of interest (ROI). Therefore, one dentist manually cropped the images from the panoramic radiographs from the right to left end of both the maxillary permanent canine germs horizontally, and from the uppermost point of both maxillary permanent canine tooth germs to the mandibular anterior alveolar bone level vertically. The images were then saved as JPEG files (Figure 1).

#### 2.3.2. Data Classification

The 1100 pre-processed images were divided into two groups. Dataset 1 was used to train the network and validate the performance of the trained model, and it consisted of 1000 images (500 images from the experimental group and 500 images from the control group). The remaining 100 images (50 images from the experimental group and 50 images from the control group) formed dataset 2 to compare the abilities of the deep-learning models and the human group to classify mesiodens.

#### 2.3.3. Network Pre-Training

SqueezeNet, ResNet-18, ResNet-101, and Inception-ResNet-V2 networks were used for classifying mesiodens. Since all the networks were pre-trained using over a million images through the ImageNet database, it was possible to learn the rich features of various images. The basic properties of the network used in the study are presented in Table 2.

#### 2.3.4. Five-Fold Cross-Validation and Data Augmentation

**Figure 2:**
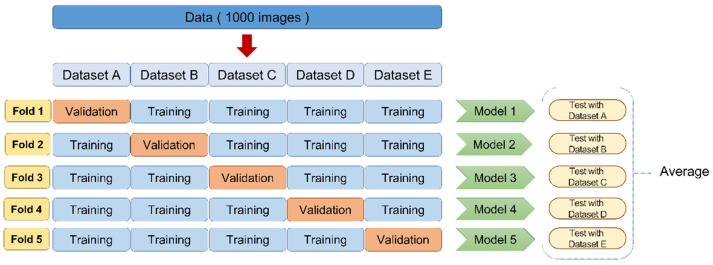
Five-fold cross-validation. The data were randomly divided into 5 groups, each consisting of 200 images. Four of these groups were used as training data and the remaining group was used as validation data. The diagnostic performance for each cross-validation set was evaluated, and the average of the five models was regarded as the estimated performance.

In the next step, five-fold cross-validation was performed. This method was used to overcome the deviation in small datasets used to train the deep-learning models for image classification (Figure 2). Dataset 1 (1000 images) was randomly divided into five groups of 200 images each. Four of these groups were used as training data, and the remaining group was used as validation data. It was carefully ensured that the same radiographs are not included in the training and validation data.

To prevent overfitting due to the small number of data samples, the amount of training data was increased through data augmentation. Training images were rotated from −7 to 7, scaled horizontally and vertically from 0.9 to 1.1, and translated horizontally and vertically from −5 to 5 pixels.

#### 2.3.5. Training Configuration

An NVIDIA Titan RTX (i9-7980XE CP, 6 GB ram) was used for training the networks with MATLAB 2019b GPU (MathWorks, Natick, MA, USA). The models were trained for up to 500 epochs using the Adam optimizer. The size of the mini-batch was 16, and the initial learning rate was 10^−4^. The training process was stopped prematurely if the validation accuracy did not increase more than 30 times to avoid overfitting of the pretrained network.

#### 2.3.6. Diagnostic Performance Evaluation

The diagnostic performance for each fold was calculated, and the average of the five-fold procedure was considered and evaluated as the final diagnostic performance of the deep-learning model for each network. The accuracy (1), precision (2), recall (3), F1-score (4), and area under the curve (AUC) values from the receiver operating characteristic (ROC) curve were used to evaluate the performance of the models.

(1) $$\mathrm{Accuracy}=\frac{TP+TN}{TP+TN+FP+FN}$$

(2) $$\mathrm{Precision}=\frac{TP}{TP+FP}$$

(3) $$\mathrm{Recall}=\frac{TP}{TP+FN}$$

(4) $$\mathrm{F1-score}=2\times \frac{Recall\times Precision}{Recall+Precision}$$

*TP*: true positive, *FP*: false positive, *FN*: false negative, and *TN*: true negative.

#### 2.3.7. Model Visualization

Using the class activation map, the area of the image that affected the decision of AI was visualized and compared with the actual mesiodens location. A class activation map was generated for each network by obtaining the weighted sum of the last convolutional features (activation maps) using the fully connected layer weights [14].

#### 2.3.8. Comparison of the Ability of Deep-Learning Models and Human Groups

To compare the abilities of the deep-learning models and human evaluators to classify mesiodens, 100 panoramic radiographs were classified in dataset 2. As human evaluators, six pediatric dentists with more than five years of clinical experience and six general dentists with less than a year of clinical experience participated in the study. Human evaluators and four deep-learning models classified dataset 2, and the times taken were measured. The accuracy, precision, recall, and F1-score for the classification results were calculated, as was the average value for each group.

#### 2.3.9. Statistical Analysis

The investigated data were analyzed using SPSS 26.0 (SPSS Inc., IBM, Chicago, IL, USA). The Kruskal–Wallis test was used to verify the statistical significance of the classification performance of deep-learning models and human evaluators.

## 3. Results

### 3.1. Classification Performance of Deep-Learning Models

**Figure 3:**
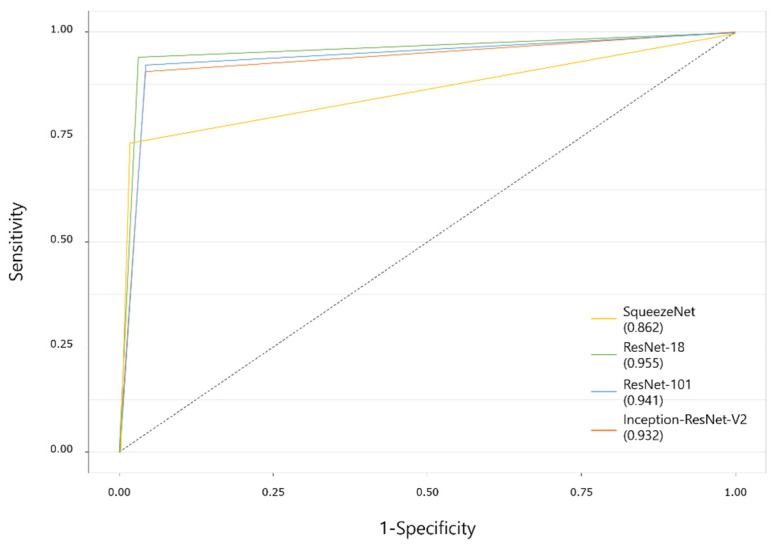
Receiver operating characteristic curves of deep-learning models on dataset 1. Numbers in parentheses show the area under the curve values.

Table 3 details the mesiodens classification performance of the deep-learning models for dataset 1. SqueezeNet showed relatively lower accuracy, precision, and F1-score than those of the other three networks. ResNet-101 and Inception-ResNet-V2 showed evenly high values in all categories. The AUC values of the deep-learning models that delivered the best performance were 0.862 for SqueezeNet, 0.955 for ResNet-18, 0.941 for ResNet-101, and 0.932 for Inception-ResNet-V2 (Figure 3).

### 3.2. Visualization of Model Classification

**Figure 4:**
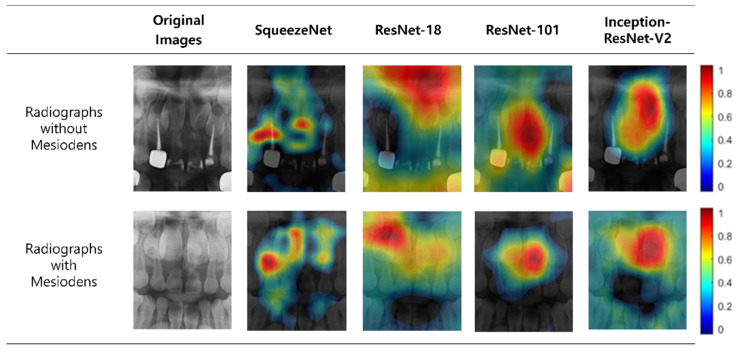
Example of the class activation maps of four deep-learning models.

To identify the image regions that affect the classification result, we represented the regions with heat maps using class activation maps. The results of these heat maps for the classification are presented in Figure 4. In images with mesiodens, the deep-learning models focused on the actual locations of the mesiodens in many cases, but there was a slight difference in focus for each network. For images without mesiodens, the networks tended to evaluate the maxillary anterior region as a whole, including permanent incisors.

### 3.3. Comparative Evaluation of the Abilities of the Deep-Learning Models and Human Groups

**Figure 5:**
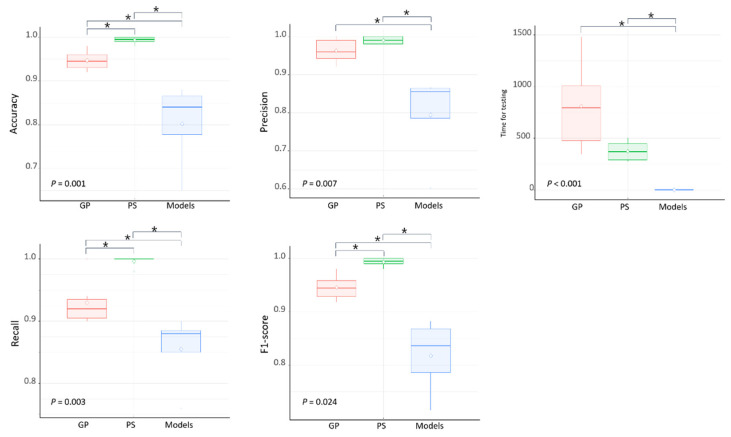
Boxplot of diagnostic performances among three groups. Kruskal–Wallis test was performed to analyze the statistical significance of specificity. GP: General Practitioners, PS: Pediatric Specialists, * *p* < 0.05; Kruskal–Wallis test.

**Figure 6:**
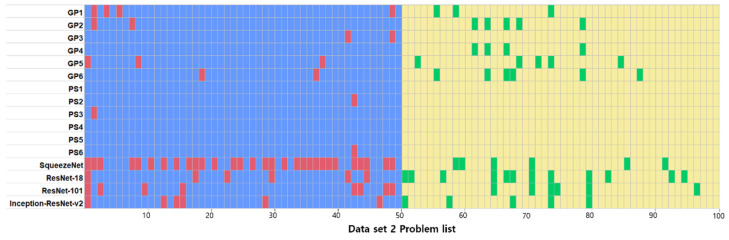
Differences in answers between human evaluators and deep-learning models on dataset 2. Blue: true positive, Red: false negative, Yellow: true negative, and Green: false positive. GP: General Practitioners, PS: Pediatric Specialists.

The classification results of the human evaluators and the deep-learning models on dataset 2 are presented in Table 4. The results of the Kruskal–Wallis test in terms of the accuracy, recall, and F1-score of the three groups of general dentists, pediatric dentists, and deep-learning models showed significant differences (*p* < 0.05, Table 4, Figure 5). Figure 6 shows the correct answer for each question according to the interpreter. The lists of incorrectly diagnosed problems tended to be similar among the deep-learning models, whereas the similarity decreased between human evaluators and deep-learning models.

## 4. Discussion

In 2017, Anthonappa et al. [8] conducted a study on the effectiveness of panoramic radiographs in identifying supernumerary teeth. According to that study, the sensitivity of supernumerary teeth classification using panoramic radiographs was 0.50. Further, a significant difference in sensitivity was confirmed between pediatric dentists (0.60) and dentists with less than one year of experience (0.39). Based on these results, the authors stated that the clinical experience of the dentist significantly influences the identification of supernumerary teeth with panoramic radiographs and that it is difficult to trust panoramic radiographs as a tool for identifying supernumerary teeth.

Recently, research on deep-learning systems has been increasing in various fields, including oral and maxillofacial radiology. Among the various deep-learning functions, the classification function is frequently used in panoramic radiographs and has been reported to perform excellently in the evaluation of various diseases [9,11,12,13]. Herein, we attempted to provide a background that can help clinicians in realizing accurate and rapid diagnosis by creating models that automatically classify mesiodens in panoramic radiographs using CNNs.

In this study, four popular pre-trained networks in Pareto frontier were applied for mesiodens classification while considering the accuracy and computational burden. Pareto frontier in the field of deep learning comprises networks that outperform other networks in terms of accuracy and prediction time [18]. Deeper networks can generally achieve higher accuracies by learning richer feature representations. However, deep networks require larger amounts of computing power and are characterized by longer prediction times when using graphic processing units (GPUs), and these are difficult to realize in average research and clinical environments. Therefore, in this study, we found the optimal pre-trained network architecture that satisfies the requirements of both accuracy and computing power for the classification of mesiodens on panoramic radiographs. The tested networks were SqueezeNet, ResNet-18, ResNet-101, and Inception-ResNet-V2.

All the deep-learning models used in this study yielded AUC values exceeding 0.85 on dataset 1. Among the four models, SqueezeNet, which has the shallowest depth and parameters, had a relatively low accuracy of 0.833. ResNet-101 and Inception-ResNet-V2, which are relatively deep networks, deliver the highest performance in most cases, although there are some differences depending on the metrics. In this study, the number and depth of parameters of the CNN and the accuracy of the model showed positive correlations, supporting the fact that the deeper the network, the higher the accuracy [19].

The CNN had the disadvantage of not being able to explain the characteristics of machine learning and decisions made by learning owing to the black-box effect [20]. McNamara et al. [21] reported that the class activation map helps solve the black-box effect by visualizing the area observed by the CNN model during object classification, and various attempts have been made using class activation maps since then in this regard. In this study, the part that plays an important role in classifying mesiodens in the panoramic radiograph was identified, using the class activation map. In most images, the network observed the actual locations of the mesiodens, but there was a slight difference in focus between all the networks. If a deep-learning model is visualized using class activation maps, it will influence humans to trust the decision made by the AI. It is possible to increase the clinical applicability of the deep-learning models by visualizing the presence and locations of the excessive number of teeth.

In this study, when the classification abilities of humans and deep-learning models were compared, the accuracy of the deep-learning models was lower than that of the humans, but the detection was significantly faster. It took an average of 811.8 s for the general dentist group and 375.5 s for the pediatric dentist group to perform evaluations on dataset 2, but the deep-learning models performed detection and classification of the entire test set within a few seconds, showing a significant difference. In a study conducted by Hiraiwa et al. [12] in 2019, the model using AlexNet and GoogleNet completed the classification of the distal root of the mandibular first molar in 163 panoramic radiographs within 9 and 11 s, with accuracies of 87.4% and 85.3%, respectively. If a deep-learning model is used as an auxiliary means for diagnosing mesiodens after taking panoramic radiographs, it would help shorten the diagnosis time of clinicians with little experience with mixed dentition panoramic radiographs. In addition, it will be possible to reduce complications due to mesiodens by increasing the probability of detecting mesiodens in advance.

In this study, the accuracies of the deep-learning models were slightly lower on dataset 1 than on new data (dataset 2) that were not used for validation. This is thought to be due to overfitting with a small number of training data. The performance of deep-learning systems typically depends on the amount of data available for training [22,23]. There was a limitation in collecting the training data because this study incorporated panoramic radiographs from a single institution. In a follow-up study, it is thought that the performance of the model can be further improved by training the network using radiographs from multiple institutions to develop a larger dataset.

This study also had some other limitations. Firstly, the dental age, which affects the difficulty of detecting mesiodens, of both the experimental group and the control group were set under the same conditions. As a result of this, the experimental group and the control group had slight differences in age and gender of the subjects. Secondly, it was difficult to determine the numbers or exact locations of mesiodens by only classifying their presence or absence in panoramic radiographs. The use of a network including object detection and classification functions, such as Single Shot MultiBox Detector, can help identify the distribution of supernumerary teeth. Lastly, in pediatric patients with primary dentition, intraoral radiographs are often taken instead of panoramic radiographs to minimize unnecessary radiation dose. A follow-up study using intraoral radiographs of the maxillary anterior region for an early detection of supernumerary teeth is necessary.

## 5. Conclusions

The deep-learning network models used in this study delivered high accuracy in classifying the presence of mesiodens in the mixed dentition panoramic radiographs. Although there was a difference in focus on class activation maps between the networks, the actual locations of the mesiodens and their surrounding teeth were typically observed. The results of this study are expected to help clinicians make decisions by automatically classifying these teeth on panoramic radiographs taken during the eruption period of the maxillary anterior teeth, thereby reducing complications that may arise from failure of early detection of mesiodens.

## Figures and Tables

**Table 1:** Demographic data of subjects in this study.

Characteristics	Patients without Mesiodens (*n* = 550)	Patients with Mesiodens (*n*= 550)
**Mean Age (SD)**	7.2 (1.3)	6.8 (1.0)
**Sex**		
Female	253	126
Male	297	424
**Hellman’s stages**		
IIA ^1^	130	130
IIC ^2^	385	385
IIIA ^3^	35	35

^1^ IIA: Completion of primary occlusion, ^2^ IIC: Eruptive phase of permanent first molars or incisors, ^3^ IIIA: Eruption of permanent first molars or incisors completed.

**Table 2:** Properties of pre-trained convolutional neural networks (CNNs).

Network Model	Depth	Size (MB)	Parameter (Millions)	Input Image Size
SqueezeNet	18	4.6	1.2	227 × 227 × 3
ResNet-18	18	44.0	11.7	224 × 224 × 3
ResNet-101	101	167.0	44.6	224 × 224 × 3
Inception-ResNet-V2	164	209.0	55.9	299 × 299 × 3

**Table 3:** Performances of deep-learning models on dataset 1.

Pre-Trained Network	Accuracy	Precision	Recall	F1-Score
SqueezeNet	0.833	0.779	0.960	0.855
ResNet-18	0.914	0.883	0.958	0.918
ResNet-101	0.927	0.911	0.948	0.928
Inception-ResNet-V2	0.924	0.916	0.934	0.925

**Table 4:** Comparison of diagnostic performances of deep-learning models and human groups.

	Accuracy	Precision	Recall	F1-Score	Time for Testing (s)
GP	0.95	0.96	0.90	0.93	811.8 ± 426.1
PS	0.99	0.99	1.00	0.93	375.5 ± 95.9
SqueezeNet	0.65	0.60	0.88	0.72	1.5 ± 1.4
ResNet-18	0.82	0.86	0.76	0.81	
ResNet-101	0.86	0.85	0.88	0.86	
Inception-ResNet-V2	0.88	0.87	0.90	0.88	

GP: General Practitioners, PS: Pediatric Specialists.

## Data Availability

Restrictions apply to the availability of these data. Data used in this study were obtained from Pusan National University Dental Hospital and are available with the permission of the Institutional Review Board of Pusan National University Dental Hospital, Pusan National University, Dental Research Institute.
